# Anodal and cathodal transcranial direct current stimulations of prefrontal cortex in a rodent model of Alzheimer’s disease

**DOI:** 10.3389/fnagi.2022.968451

**Published:** 2022-08-23

**Authors:** Mengsi Duan, Zhiqiang Meng, Dong Yuan, Yunfan Zhang, Tao Tang, Zhuangfei Chen, Yu Fu

**Affiliations:** ^1^Medical School, Kunming University of Science & Technology, Kunming, China; ^2^Shenzhen Key Laboratory of Drug Addiction, The Brain Cognition and Brain Disease Institute (BCBDI), Shenzhen Institute of Advanced Technology, Chinese Academy of Sciences, Shenzhen, China; ^3^CAS Key Laboratory of Brain Connectome and Manipulation, Shenzhen Institute of Advanced Technology, Chinese Academy of Sciences, Shenzhen, China; ^4^Shenzhen-Hong Kong Institute of Brain Science-Shenzhen Fundamental Research Institutions, Shenzhen, China

**Keywords:** tDCS, spatial cognition, EEG, Alzheimer’s disease, mouse

## Abstract

Alzheimer’s disease (AD) is a leading cause of dementia in the elderly, with no effective treatment currently available. Transcranial direct current stimulation (tDCS), a non-drug and non-invasive therapy, has been testified efficient in cognitive enhancement. This study aims to examine the effects of tDCS on brain function in a mouse model of AD. The amyloid precursor protein (APP) and presenilin 1 (PS1) transgenic mice (7–8 months old) were subjected to 20-min anodal and cathodal tDCS (atDCS and ctDCS; 300 μA, 3.12 mA/cm^2^) for continuous five days. tDCS was applied on the left frontal skull of the animals, targeting on their prefrontal cortex (PFC). Behavioral performances were assessed by open-field, Y-maze, Barnes maze and T-maze paradigms; and their PFC electroencephalogram (EEG) activities were recorded under spontaneous state and during Y-maze performance. Behaviorally, atDCS and ctDCS improved spatial learning and/or memory in AD mice without affecting their general locomotion and anxiety-like behaviors, but the effects depended on the testing paradigms. Interestingly, the memory improvements were accompanied by decreased PFC EEG delta (2–4 Hz) and increased EEG gamma (20–100 Hz) activities when the animals needed memory retrieval during task performance. The decreased EEG delta activities could also be observed in animals under spontaneous state. Specifically, atDCS increased PFC EEG activity in the alpha band (8–12 Hz) for spontaneous state, whereas ctDCS increased that in alpha-beta band (8–20 Hz) for task-related state. In addition, some EEG changes after ctDCS could be found in other cortical regions except PFC. These data indicate that tDCS can reverse the situation of slower brain activity in AD mice, which may further lead to cognitive improvement. Our work highlights the potential clinical use of tDCS to restore neural network activity and improve cognition in AD.

## Introduction

Alzheimer’s disease (AD) is the most common cause of dementia in the elderly. Dementia is now the seventh leading cause of mortality in the world (Patterson, 2018). However, to date, there are no effective treatments that can slow or cure the disease. Many clinical trials and drug developments in AD have resulted in disappointing outcomes (Anand et al., 2014; Abbott and Dolgin, 2016), suggesting a need for new approaches used in AD treatments.

Transcranial direct current stimulation (tDCS) is a non-drug and non-invasive neuromodulation technique. Accumulating studies have verified the safety of the technique (Bikson et al., 2016) as well as the long-term after-effects up to 2 months (Yang et al., 2019). Through electrodes placed on the scalp, tDCS can modulate neural activity by delivering a constant and weak direct current to specific regions of the brain. It regulates cell membrane potential depolarization and hyperpolarization and thereby alters activity of neurons and excitability of cerebral cortex (Nitsche et al., 2003, 2008; Das et al., 2016). Anodal tDCS (atDCS) refers to the application of positive current whereas the cathodal (ctDCS) applies negative current to the target. It has been indicated that tDCS changes the neural state by modulating the neural firing rate (Creutzfeldt et al., 1962), which is increased by delivering atDCS and decreased by delivering ctDCS (Nitsche and Paulus, 2000).

Most clinical studies in patients show that tDCS has benefits for healthy aging and mild cognitive impairment (MCI)—and AD-associated cognitive declines, but a few studies show inconsistent results. For both healthy and pathological aging, the benefits of tDCS (most from atDCS) were reported in many cognitive functions, such as working memory, situational memory, location memory, and so on (Hsu et al., 2015; Prehn and Floel, 2015). However, there are a few reports describing neutral or negative effects of tDCS on cognition (Leach et al., 2016, 2019). For example, tDCS did not significantly improve verbal memory function in AD patients (Bystad et al., 2016); and tDCS blocked cognitive benefits of cognitive training on executive function and episodic memory in MCI adults (Das et al., 2019).

The dorsolateral prefrontal cortex (DLPFC) was a common target site of tDCS in clinical studies (Boggio et al., 2009; Cotelli et al., 2014; Khedr et al., 2014; Cespon et al., 2019; Im et al., 2019). tDCS that was performed over the bilateral DLPFC improved or stabilized cognition in AD patients (Im et al., 2019), and tDCS that was applied over the left DLPFC could not only improve cognitive function but also reduce the P300 event-related potential latency (Khedr et al., 2014). Thus, it would be valuable to examine the possible mechanism of tDCS over the DLPFC that is involved in improving the AD brain.

By contrast, there are limited data available on basic research in animals, which also show inconsistent results (Nardone et al., 2015). For example, in rodent models of AD, tDCS was effective to improve spatial learning and memory and attenuate amyloid beta (Aß) levels, and the improvement could be maintained for a long time (Yu et al., 2014, 2015; Yang et al., 2019; Luo et al., 2020). However, there is another study showing that tDCS did not improve memory deficits or alter pathological hallmarks of AD (Gondard et al., 2019). Together with clinical data, the role of tDCS in augmenting cognitive function still need further studies (Chang et al., 2018; Das et al., 2019).

On the other side, the underlying mechanism of tDCS still remains unclear, especially that for AD is in its infancy (Chang et al., 2018). Recently, there are clinical studies exploring the effect of tDCS on brain activity in AD patients by using electroencephalogram (EEG) (Yang et al., 2021). EEG is a sensitive measure to assess neurophysiological changes occurring in physiological and pathological aging (Tatti et al., 2016). EEG patterns were abnormal in the brains of AD patients, and atDCS could reverse the patterns and modulate cortical activity (Marceglia et al., 2016; Cespon et al., 2019; Gangemi et al., 2020). For basic research, the AD animal models also exhibit abnormal EEG activity, such as a prominent EEG slowing (Dringenberg, 2000; Jyoti et al., 2010; Platt et al., 2011; Schneider et al., 2014; Del Percio et al., 2018; Hidisoglu et al., 2018). However, how tDCS affects the EEG pattern has been rarely reported in the AD animals.

In this study, we conducted a series of behavioral and EEG experiments to explore the possible role of tDCS and the related neural mechanism in a mouse model of AD. We applied tDCS over the prefrontal cortex (PFC) of animals, which agrees to most clinical studies that target the DLPFC for the stimulation (Cespon et al., 2019; Im et al., 2019). We speculated that if tDCS showed behavioral improvement, it might improve high frequency EEG activity in the AD brain. Importantly, unlike most previous studies where only atDCS was tested, we would also examine the role of ctDCS in the AD brain. Because ctDCS tends to reduce cortical excitation and the AD brain shows aberrant increases in network excitation (Palop et al., 2007), we hypothesized ctDCS would be effective in the AD brain.

## Materials and methods

### Animals

Breeding pairs of Amyloid precursor protein (APP)/presenilin-1 (PS1) double transgenic male mice (B6C3-TgAPPswe, PSEN1dE985DboJNju; abbreviated to AD mice) were obtained from Guangdong Medical Laboratory Animal Center (license number SCXK [Yue] 2018-0002). The transgenic mice express a chimeric mouse/human APP (Mo/HuAPP695swe) and a mutant human PS1-dE9 directed to central nervous system (CNS) neurons. Genotypes were confirmed by polymerase chain reaction (PCR) genotyping using genomic DNA extracted from tail tissue samples. The strain of mice develops beta-amyloid (Aβ) deposits in the brain by 6–7 months of age. Both male and female mice were used in this study, and they were 7–8 months old. During the experimental period, mice were housed in groups of 1–3 animals in plastic cages (30 cm × 18 cm × 14 cm) under constant temperature (23 ± 1°C) and stable humidity with a natural light-dark cycle. They had free access to food and water. The experimental and animal care procedures were performed according to the guidelines for the National Care and Use of Animals and approved by the National Animal Research Authority. Furthermore, all experiments were approved by the Animal Care and Ethics Committee of Kunming University of Science and Technology (approval No. 20190001).

### Experimental design

**FIGURE 1:**
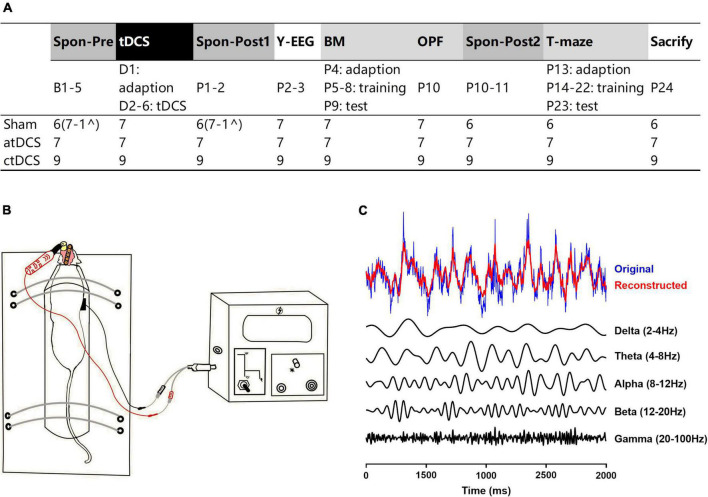
Experimental design. **(A)** Schematic of study design and group compositions with number of animals used in different paradigms. Spontaneous EEG activities were recorded in three groups of AD mice (atDCS, anodal tDCS; ctDCS, cathodal tDCS; and Sham, sham stimulation) for three times: one time before (Spon-Pre) and two times after tDCS (Spon-Post1 and Spon-Post2). Task-related EEG activities were recorded during Y-maze performance (Y-EEG). Cognitive performance was assessed by Barnes maze (BM), open-field (OPF), and T-maze tasks. Animals were finally sacrificed for further histological examination. **(B)** Animals were implanted by an epicranial plastic tube for filling with saline as active electrode for tDCS and by metal electrodes for EEG recording. **(C)** EEG segment was filtered for five frequency bands (delta-gamma) for power analysis. Original EEG and reconstructed EEG (a superposition of five frequency-filtered bands) were showed. B1-5, D1-5, and P1-24: days before, during and after tDCS. ^Electrodes from an animal were broken during Spon-post2 session, so the spontaneous EEG data for this animal were excluded from Spon-pre and Spon-post2 sessions.

The AD mice were randomly divided into three groups: (1) atDCS group with anodal tDCS; (2) ctDCS group with cathodal tDCS; and (3) sham group with sham stimulation (Figure 1A). We used a protocol of tDCS similar to that described previously (Cambiaghi et al., 2010). Mice were implanted with an epicranial plastic tube for filling with saline as active electrode prior to stimulation. In the present study, animals were meanwhile implanted with metal electrodes for EEG recording (Figure 1B). After the surgery of electrode implantation, mice were allowed at least one week for recovery. In addition, all mice were allowed 3 days for habituation in recording and stimulation conditions before the beginning of the experimental routine.

The experimental routine was diagrammed in the figure (Figure 1A). tDCS was given for five consecutive days, with one day prior for adaption for the whole stimulation protocol (with no current stimulation). Spontaneous EEG activity was acquired for three times: one time before (Spon-Pre) and two times after (Spon-Post1 and Spon-Post2) tDCS. EEG activity during Y-maze performance (Y-EEG) was acquired for one time after tDCS. In addition, behavioral performance was assessed using the following tasks: Barnes maze (BM), open field (OPF), and T-maze. Mice were finally sacrificed and the brains were removed for further histological examination (right brains) and enzyme-linked immunosorbent assay (ELISA) procedure (left brains). All experiments were completed within 1 month after the first tDCS. Animal numbers for each group were detailed in the figure (Figure 1A). It is noted that for spontaneous EEG recording, the electrodes from one animal could not collect EEG signals during the third recording, so data from the first and second recordings were excluded out for this animal.

### Surgery

Surgery was performed under pentobarbital anesthesia (80 mg/kg, i.p.; dissolved in saline, 10 mg/ml, Merck, Darmstadt, Germany). After a midline scalp incision, the scalp and underlying tissues were removed. An epicranial plastic tube (inner diameter: 3.5 mm) was first implanted with its center positioned over the left PFC (AP: +2.95 mm, ML: –1.5 mm). The tube was tightly glued onto the skull by modified acrylate adhesive. In addition, four burr holes were drilled in the skull for implanting EEG electrodes. One twisted pairs of perfluoroalkoxy (PFA)-coated stainless steel wires (diameter: 0.002″, A-M systems, WA, United States) were implanted in the brain through one hole, serving as an EEG recording electrode, to record the right PFC (AP: +2.80 mm, ML: +1.0 mm, DV: –0.5 mm from dura). A stainless-steel watch screw (M1.0 × L2.0 mm, RWD) was placed in contact with the dura through one hole, serving as the left cortex (Ctx) recording electrode (AP: –3.8 mm, ML: –2.5 mm). Through the other two holes, two stainless-steel watch screws were also placed in contact with the dura above the left olfactory bulb and central cerebellum, serving as reference and ground electrodes, respectively. All electrodes were attached to male pins that were secured in a rectangular pin array. Finally, all electrodes together with the epicranial plastic tube were secured with dental acrylic.

### Transcranial direct current stimulations

Before tDCS, the animal was restrained on a self-made fixed table in an awake state, keeping its head and torso still (Figure 1B). The animal remained consciousness during the stimulation to prevent the interaction between current stimulation effect and anesthetic drugs. For tDCS, the epicranial plastic tube was filled with saline, serving as active electrode (round, diameter: 3.5 mm). The counter electrode was a saline-soaked sponge (round, diameter: 12 mm) applied over the ventral thorax. Here, the electrodes of saline and saline-soaked sponge were chosen instead of metal electrodes, mainly because the latter can be polarized by the direct current of tDCS. Both atDCS and ctDCS were applied at a current intensity of 300 μA for 20 min by a constant current stimulator (Cerebooster, Droian, Hangzhou Zhuo An Zi network technology Co. Ltd.). The current intensity corresponds to a density of 3.12 mA/cm^2^ (300 μA/0.096 cm^2^). The current flow changed linearly within 10 s at the beginning and the end of tDCS to avoid a stimulation break effect from switching it on and off directly. In addition, the operation of sham group was the same as that of tDCS groups but with no current applied. tDCS was conducted for five consecutive days.

### Electroencephalogram recording protocol

EEG signals were collected by EEG acquisition system, which consisted of an RHD2132 amplifier, RHD2000 USB interface board, and RHD2000 Interface GUI Software (Intan Technologies, Los Angeles, CA, United States). Data were acquired at a sampling rate of 1,000 Hz. The rectangular pin array of electrodes on each animal was connected by a cable to the amplifier, then to the interface board, and finally to the computer. The cable was suspended by a helium balloon to allow the animal free movement. EEG recording was performed in a shielding cage (80 cm × 70 cm × 100 cm, width × length × height). The animal behaviors in the cage were monitored with a ceiling-mounted camera. The video signals were displayed and saved by video-recording software.

Spontaneous and task-related EEG signals were collected in the shielding cage. Spontaneous EEG signals were acquired three times for each animal. Each time lasted for 30 min and the animal was under spontaneous and awake state. A new home cage was placed in the shielding cage, and the floor of home cage was covered with sawdust. This condition is similar to their conditions of daily living. Task-related EEG signals were acquired one time for each animal during Y-maze performance. The task consisted of two trials: 10-min training trial and 5-min testing trial. EEG signals were acquired for both trials. For this task, the shielding cage was decorated with a curtain inside. Several visual spatial cues, such as black square, yellow circular grating, and white triangle, were suspended on the curtain. In addition, the camera, amplifier, and exposed wood of the cage were used as spatial cues. A Y-maze was placed in the shielding cage, and the floor of the maze was covered with sawdust, which was mixed after each trial to equate differential olfactory stimuli. Please refer to the section of Behavioral tests for details of the Y-maze task.

### Behavioral paradigms

Spatial learning and memory were assessed in animals after tDCS (Figure 1A). Short-term recognition memory was evaluated by two-trial Y-maze test, long-term learning and memory were by Barnes maze (BM) test, and working memory was by T-maze test. In addition, general locomotor activity and anxiety-like behavior of animals were assessed by open field (OPF) test. During behavioral tests, a video-monitoring system (KEmaze, China KEW Basis) was simultaneously used to obtain and analyze the trajectories of animal.

The Y-maze was made of black PVC board, and consisted of three arms with an angle of 120° between adjacent arms. There was 1–2 differential visual cues placed on the walls of each arm. Each arm was 8 cm × 30 cm × 15 cm (width × length × height). The three identical arms were randomly designated for each animal: (1) start arm, in which the animal started to explore (always open); (2) novel arm, which was blocked during the training trial, but open during the testing trial; and (3) other arm (always open). The Y-maze task consisted of 10-min training trial, 5-min testing trial (retrieval test), and 1-h inter-trial interval (ITI) phase. The animal was returned to its home cage during the ITI phase. During training, the animal was allowed to explore the start and other arms, with the novel arm blocked. During testing, the animal was allowed to explore all three arms. The percentage of time spent in the novel and other arms and the percentage of number of arm visits were calculated for each animal. Data in the start arm were excluded to avoid the stimuli of placing the animal in the maze. The first choice for novel arm was also recorded. These data in testing trial were as spatial recognition memory indices. In addition, the total number of arm visits in the maze was counted for each animal as a locomotor activity index for both training and testing trials.

The BM maze consisted of a round platform and a stainless steel bracket. The platform was 90 cm high from floor. It was made of white PVC board and the diameter was 90 cm. The platform contained 20 holes, each 5 cm in diameter, equally distributing around the platform. During the experiment, one hole was randomly assigned to be the target hole for each animal. There was an escape box in the target hole, which was communicated with the platform through transparent plastic tunnels. The escape box cannot be seen from the platform.

The BM task included habituation, acquisition and probe phases. During habituation (day 1), the animal was placed on the center of the platform, and two bright lights were turning on as an aversive stimulus. The animal was guided to the target hole, and once it was in the escape box, the lights were turned off and the animal was kept inside for 2 min. The acquisition (days 2–5) phase lasted for 4 days and each day included four trials. Each trial lasted for 3 min with an ITI of 15 min. In each trial, the animal was placed on the center of the platform and the lights were turned on. The latency to find the target hole was recorded for each animal. If the animal did not find the target hole within 3 min, it was placed at the entrance of the target hole for 1 min and was then returned to its home cage. The probe test (day 6) was conducted 24 h after the last acquisition trial. The escape box was taken out, and the animal was allowed to explore the maze for 90 s. The latency to find the target hole in the first place was recorded. In addition, the total ambulation and speed on the platform were calculated automatically by the software. During the task, the platform was rotated after each trial for each animal. The location of the target hole for each animal was kept in the same orientation. The platform was cleaned with 75% alcohol and then with dry paper towel after each trial.

The OPF box was made of PVC board, with gray walls and white floor. The box was square with 50 cm × 50 cm × 30 cm (width × length × height). The animal was placed into the box with its head toward one wall and then allowed to explore the box for 5 min. The total ambulation and speed in the box were calculated automatically by the software. The ambulation and time spent in the central area (25 cm × 25 cm, width × length) of the box were also calculated. In addition, the times of rearing and the number of fecal droppings (defecation rate) were recorded for each animal. The box was cleaned after each test.

The T-shaped maze was made of black PVC board and consisted of a start arm and two choice arms. The start arm was 40 cm × 8 cm × 15 cm (length × width × height) and the choice arm was 30 cm × 8 cm × 15 cm (length × width × height). A recessed black plastic food cup (4 cm in diameter, 1 cm in depth) was placed on the floor at the end of each choice arm. The maze was enclosed by a curtain with some visual spatial cues suspended on it. The animal in the maze could see the cues outside the maze.

The T-maze task included habituation, training and testing phases. During habituation (day 1), there were two pieces of peanuts in both food cups, and the animal was placed in the start arm and allowed to explore the maze for 10 min. The animal was returned to its home cage after habituation. During training (days 2–10), there was two pieces of peanuts only in one choice arm designed as correct arm for each animal. The training phase lasted for 9 days and each day included five trials. Each trial began by placing the animal in the start arm with its head toward the wall, and ended when the animal entered the correct arm and ate the peanut. If the animal did not choose either arm or correct arm within 2 min, it was returned to its home cage. The ITI varied randomly within 40–60 s. The testing phase (day 11) included only one trial and there was nothing in both food cups. The trial began by placing the animal in the start arm and ended when the animal entered one choice arm. During the experiment, one choice arm was randomly assigned to be correct arm for each animal, but the correct arm and orientation of the maze were kept stable for each animal. Correct rate for every day and mean correct rate for the total training phase were calculated for each animal. Correct rate for the testing day was also calculated for the stimulation group. The floor of the maze was covered with sawdust, which was mixed after each individual trial to equate differential olfactory stimuli.

### Histological examination (right brains) and enzyme-linked immunosorbent assay procedure (left brains)

After all experiments, animals were anesthetized with pentobarbital sodium (80 mg/kg, i.p.). Electrolytic lesions were first made by applying an anode direct current (60 mA, 2.5 min) to the PFC electrode to mark the EEG recording location. The animals were then transcardially perfused with saline. Brains of mice were removed after decapitation and dissected on an ice-cold plate to isolate: (1) the right brains for histologically confirming Aβ plagues and EEG recording locations; and (2) the left hippocampal tissues for ELISA experiment. Followed (1), the right brains were post-fixed in 4% paraformaldehyde (PFA) at 4°C for 48 h, dehydrated twice in 30% sucrose with PBS at 4°C, and then stored in 4°C refrigerator until further use. Followed (2), the left hippocampal tissues were initially stored in a liquid nitrogen tank and then in –80°C refrigerator until further use.

#### Confirming Aβ plagues

The right brain tissues were embedding with optimal cutting temperature (OCT) compound and frozen sectioned at 8-μm thickness. The sections were stained with indirect immunofluorescence staining method. The sections were washed three times each with 1 × PBS for 3 min, placed in sodium citrate buffer solution (10 mM, 0.05% Tween-20, pH 6.0), heated in a microwave oven, and cooled to room temperature for antigen repair. They were then blocked in a 1 × PBS containing 3% BSA and 10% goat serum for 1.5 h. After removing the blocking buffer, the brain tissues were encircled with an immunohistochemistry pen. The primary antibody (ß-amyloid (D54D2) XP^®^ Rabbit mAb #8243) was added into the circle for incubating overnight in a wet box at 4°C. The antibody concentration was 1:600 and the antibody diluent was 1% BSA and 0.3% Triton in 1 × PBS. After incubation, the sections were washed three times each with 1 × PBS for 3 min. The fluorescent secondary antibody (Rabbit red) was added to incubate for 1 h. The antibody concentration was 1:1,000 and the antibody diluent was 1% BSA and 0.3% Triton in 1 × PBS. Finally, the slices were washed three times and then followed by mounting with 4’,6-diamidino-2-phenylindole (DAPI) staining and cover-slipping on the microscope slides. Images were acquired by using a confocal microscope (Olympus VS120).

#### Confirming prefrontal cortex electroencephalogram recording locations

Followed the section “Confirming Aβ plagues,” the brain tissues including PFC electrode locations was also exanimated, as previously reported (Fu et al., 2019). Data were excluded from any mice in which the recording locations were misplaced.

#### Enzyme-linked immunosorbent assay

The left hippocampal tissues were homogenized in RIPA buffer (150 mM NaCl, 1.0% NP-40, 0.5% sodium deoxycholate, 0.1% SDS, 50 mM Tris-HCL, pH 8.0) containing protease and phosphatase inhibitors (Thermo Scientific, Rockford, IL, United States) for protein extraction. Homogenates were then centrifuged at 3,000 rpm for 20 min at 4°C. The supernatants were collected and stored at –80°C for ELISA. The levels of soluble Aβ of the tissues were determined by a total human Aβ ELISA kit (EH025-96; ExCell Biology, Shanghai, China) in accordance with the manufacturer’s protocols. Absorbance was measured at 450 nm using a 96-well plate reader. The concentrations of total Aβ were calculated from standard curves, and data were expressed as pg/ml.

### Data analysis

As described earlier (Fu et al., 2019), EEG signals were off-line analyzed by MATLAB. EEGs were recorded by the Intan system as rhd data files. For frequency band power analysis, each rhd data file was first separated into segments, with each segment comprised of 1,024 sample points. The segments were filtered for the following EEG frequency bands (with no 50 Hz notch filter): (1) delta: 2–4 Hz, (2) theta: 4–8 Hz, (3) alpha: 8–12 Hz, (4) beta: 12–20 Hz, and (5) gamma: 20–100 Hz (Figure 1C). There is large variability for the definition of frequency bands for the EEG rhythms (Newson and Thiagarajan, 2018). In this study, the frequency range for each band was in keeping with our previous works on aging (Fu et al., 2008, 2019; Zhou et al., 2021). For each frequency band, the absolute power of each segment was calculated as: *P* = Σχ^2^/1,024. The relative power (RP) was calculated as the percentage of power relative to the total power of all frequency bands.

Data with normal distribution were expressed as means ± SEM, and data with non-normal distribution were shown as medians and range (min to max). For normally distributed data, paired-samples T test was used for paired samples; one-way ANOVA and repeated-measure ANOVA were used for independent samples or repeat measurements, respectively. For non-normal distribution data, non-parametric analyses were performed. Wilcoxon matched-pairs signed rank test was used for paired samples and Friedman test were used for repeat measurements, respectively. *P* values smaller than 0.05, 0.01, and 0.001 were considered statistically significant, highly significant, and very highly significant, respectively.

## Results

### Transcranial direct current stimulations decreased spontaneous delta electroencephalogram activity in prefrontal cortex

**FIGURE 2:**
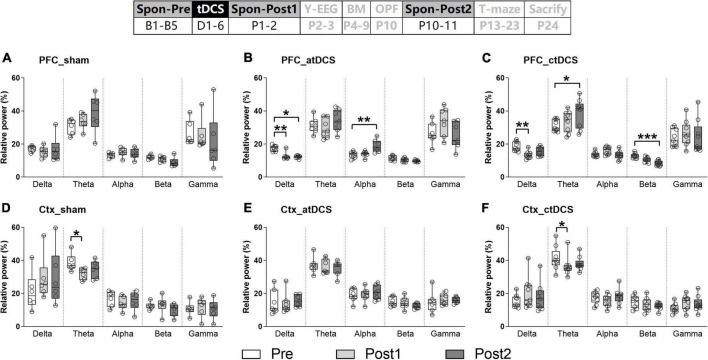
Effects of tDCS on relative power in five EEG frequency bands in AD mice under spontaneous state. EEGs were recorded from the right prefrontal cortex [PFC **(A–C)**] and the left parietal cortex [Ctx **(D–F)**; as a control]. The time point of the recordings relative to tDCS were showed (upper panel). *P < 0.05, **P < 0.01, and ***P < 0.001 compared with relative power before stimulation (Spon-Pre).

Spontaneous EEG activity was recoded once before and twice after the stimulation. The most obvious EEG changes were found in the PFC (Figure 2). For both atDCS and ctDCS, the PFC delta EEG activity showed significant decrease after the stimulation (Friedman test; χ^2^ = 11.14 and 12.67 for atDCS and ctDCS, respectively, *P* < 0.01 for both) (Figures 2B,C). The decrease could be observed in a short time (Post1, 1–2 days after tDCS) and/or in a long time (Post2, 10–11 days after tDCS) (please refer to the figure for the detailed significances from *post-hoc* pairwise comparisons; the same below). Specifically, atDCS significantly increased EEG activity in the alpha band in a long time after the stimulation (χ^2^ = 12.29, *P* < 0.01) (Figure 2B); and ctDCS significantly increased EEG activity in the theta band (χ^2^ = 6.89, *P* < 0.05) and decreased EEG activity in the beta band (χ^2^ = 18.00, *P* < 0.01) in a long time after the stimulation (Figure 2C). PFC EEG changes in five frequency bands were not significant for sham (Figure 2A).

Ctx theta EEG activity showed significant decrease in sham and ctDCS groups after the stimulation (χ^2^ = 7.00 and 6.89 for sham and ctDCS, respectively, *P* < 0.05 for both) (Figures 2D,F). As significant change was found in sham group, it may because theta EEG activity in the Ctx was affected by conditions of the recording day. In addition, EEG changes in the other frequency bands were not significant in this region (Figures 2D–F).

### Cathodal transcranial direct current stimulations improved spatial recognition memory and induced much prefrontal cortex electroencephalogram difference during Y-maze performance

**FIGURE 3:**
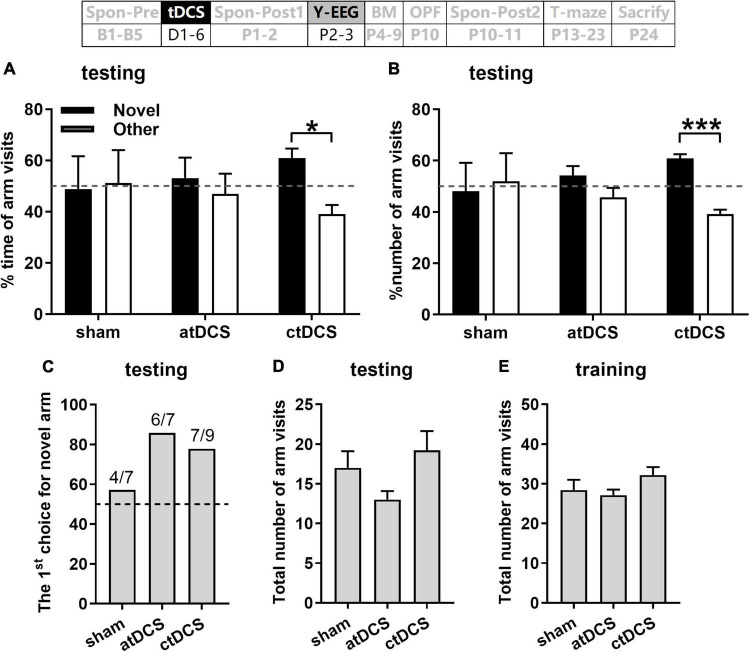
Effects of tDCS on spatial recognition memory in Y-maze task. Percentages of time spent **(A)** and number visits **(B)** to novel and other arms, and the first choice for novel arm **(C)** during 5-min retrieval trial. Total arm visits during the retrieval trial **(D)** and the 10-min training trial **(E)**. Dashed lines indicate chance level (50%). The time point of the Y-maze task relative to tDCS was also showed (upper panel). *P < 0.05 and ***P < 0.001 compared between arms.

Behaviorally, the AD animals in ctDCS group could differentiate the novel arm from the other arm in the Y-maze task. They showed significantly higher percentage time and number in the novel arm than in the other arm [Paired *t*-test; *t*(8) = 3.03 and 6.34, *P* < 0.05 and 0.001 for time and number, respectively] (Figures 3A,B). In contrast, animals in sham and atDCS groups showed similar percentage time and number in both arms (*P* > 0.05). It was noted that the first choice for the novel arm was higher for atDCS (85.71%, 6/7) and ctDCS (77.78%, 7/9) groups than sham group (57.14%, 4/7) (Figure 3C). In addition, the total number of arm visits either in training or testing trials showed no significant difference among three groups (*P* > 0.05) (Figures 3D,E).

**FIGURE 4:**
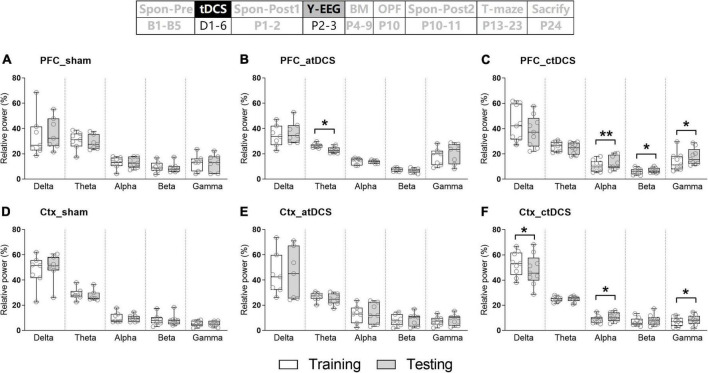
Effects of tDCS on relative power in five frequency bands in mice during Y-maze performance. EEGs were recorded from the right prefrontal cortex [PFC **(A–C)**] and the left parietal cortex [Ctx **(D–F)**]. Training: EEGs during the training trial; and Testing: EEGs during the retrieval trial. The time point of the recording relative to tDCS was also showed (upper panel). *P < 0.05 and **P < 0.01 compared between trials.

EEG activities in five frequency bands were first compared between training and testing trials for each stimulation group. It is interesting that there was much EEG difference for ctDCS group (Figure 4). PFC EEG activities in the alpha-gamma bands were significantly higher in testing trials than those in training trials (Wilcoxon test; *Z* = 2.67, 2.19 and 2.43, *P* < 0.01, 0.05 and 0.05 for alpha-gamma, respectively; the same below) (Figure 4C). Ctx EEG activities in the alpha and gamma bands were significantly higher and that in the delta band was significantly lower in testing trials than those in training trials (*Z* = 2.19, 2.19, and –2.07 for alpha, gamma, and delta, respectively, *P* < 0.05 for all) (Figure 4F). For atDCS group, PFC EEG activity only in the theta band was significantly lower in testing trials than that in training trials (*Z* = –2.20, *P* < 0.05) (Figures 4B,E). In contrast, for sham group, there was no significant EEG difference between the two trials in any frequency band (Figures 4A,D).

**FIGURE 5:**
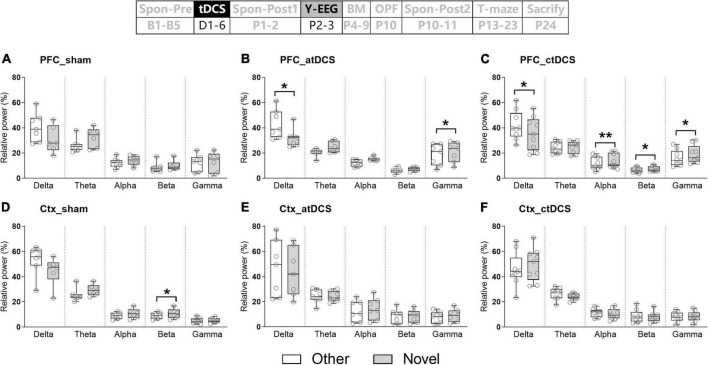
Effects of tDCS on relative power in five frequency bands in mice during Y-maze performance. EEGs were recorded from the right prefrontal cortex [PFC **(A–C)**] and the left parietal cortex [Ctx **(D–F)**]. Other: EEGs in the other arm of the maze during the retrieval trial; and Novel: EEGs in the novel arm of the maze during the retrieval trial. The time point of the recording relative to tDCS was also showed (upper panel). *P < 0.05 and **P < 0.01 compared between arms.

In addition, EEG activities in five frequency bands were compared between novel and other arms in testing trials (Figure 5). Similarly, there was much EEG difference for ctDCS group. PFC EEG activity in the delta band was significantly lower (*Z* = –2.43, *P* < 0.05) and those in the alpha-gamma bands were significantly higher (*Z* = 2.67, 2.31, and 2.55, *P* < 0.01, 0.05, and 0.05 for alpha-gamma, respectively) in the novel arm than those in the other arm (Figure 5C). In addition, for atDCS group, PFC EEG activities in the delta (*Z* = –2.20, *P* < 0.05) and gamma (*Z* = 2.37, *P* < 0.05) bands showed similar differences as those in ctDCS group (Figure 5B). For sham group, Ctx EEG activity in the beta band was significantly higher in the novel arm than that in the other arm (*Z* = 1.99, *P* < 0.05) (Figure 5D). EEG changes in five frequency bands were not significant for PFC in sham group (Figure 5A) and for Ctx in both atDCS and ctDCS groups (Figures 5E,F).

### Anodal transcranial direct current stimulations improved spatial learning and memory in Barnes maze task

**FIGURE 6:**
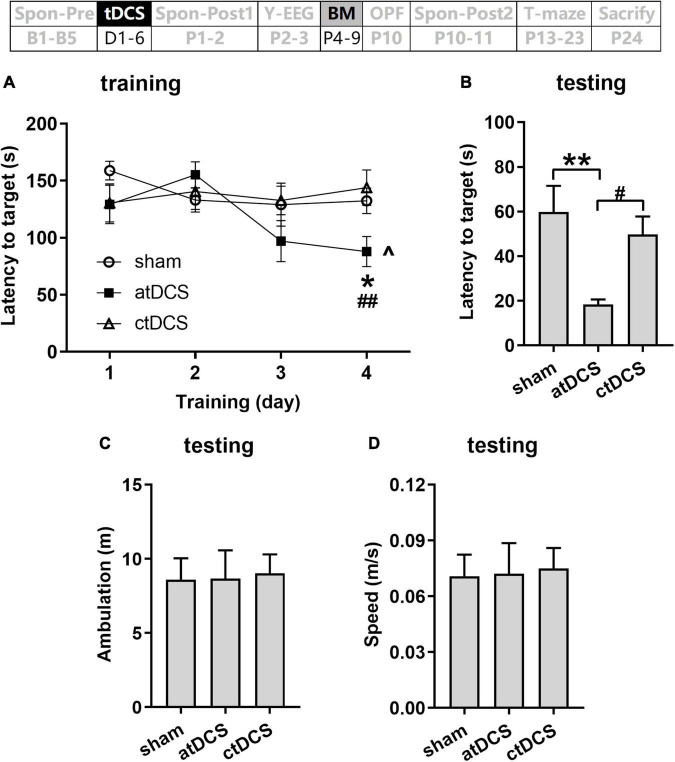
Effects of tDCS on spatial learning and memory in Barnes maze task. Latencies to target hole were showed for training **(A)** and probe **(B)** trials. Total ambulation distance **(C)** and speed **(D)** in probe trials were also showed. The time point of the task relative to tDCS was showed (upper panel). ^*P* < 0.05 compared with the first training day; *P < 0.05 and **P < 0.01 compared with sham stimulation; and ^#^*P* < 0.05 and ^##^*P* < 0.05 compared between atDCS and ctDCS.

The animals in atDCS group showed improved spatial learning ability during the 4 days training session [ANOVA-R and ANOVA-1; combined analysis: *F*(3,60) = 4.37, *P* < 0.01 for main effect of day and *F*(6,60) = 3.69, *P* < 0.01 for interaction effect of day and group; atDCS: *F*(3,18) = 8.20, *P* < 0.01; sham: *F*(3,18) = 1.61, *P* = 0.22; ctDCS: *F*(3,24) = 0.55, *P* = 0.65] (Figure 6A). They showed significantly lower latency to the target hole on the fourth day than that on the first day (*post-hoc* LSD, *P* < 0.05). In addition, on the fourth day, the atDCS group showed significantly lower latency to the target hole than both sham and ctDCS groups [ANOVA-1; *F*(2,20) = 4.43, *P* < 0.05; *post-hoc* LSD, vs sham: *P* < 0.05; vs ctDCS: *P* < 0.01].

During the testing session, atDCS group also showed improved spatial memory when compared with the other two groups [*F*(2,20) = 6.16, *P* < 0.01; *post-hoc* LSD, vs sham: *P* < 0.01; vs ctDCS: *P* < 0.05] (Figure 6B). Total ambulation distance and speed showed no significant difference among three groups (*P* > 0.05) (Figures 6C,D).

### Transcranial direct current stimulations unaffected general locomotor activity and anxiety-like behavior in open field test

**FIGURE 7:**
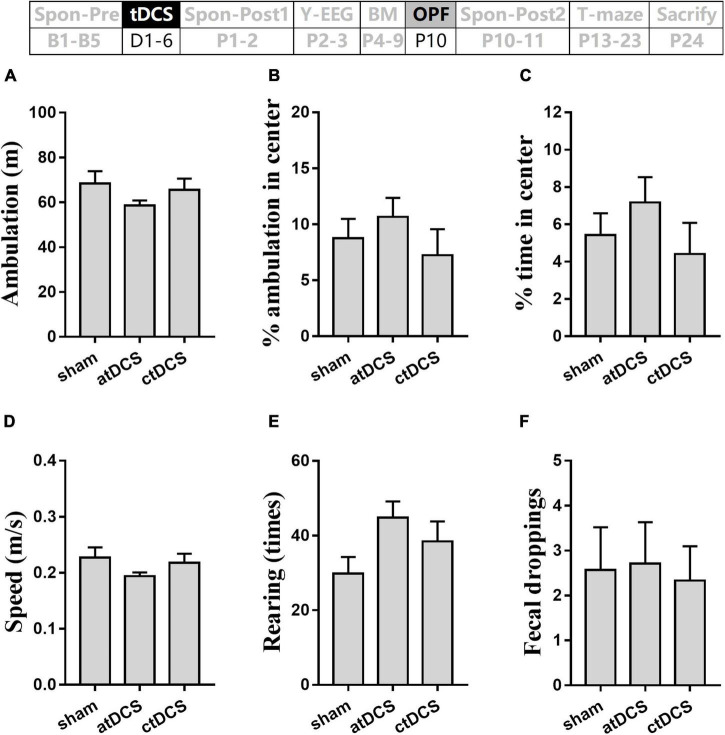
Effects of tDCS on locomotion, general-anxiety, and exploration activity in open-field test. Total ambulation distance **(A)** and speed **(D)**, percentages of ambulation **(B)** and time **(C)** in center area, times of rearing **(E)**, and number of fecal dropping **(F)** in the test were showed. The time point of the test relative to tDCS was showed (upper panel).

Total ambulation distance and speed in the OPF showed no significant difference among the three groups (*P* > 0.05) (Figures 7A,D). The percentages of ambulation and time in the center area also showed no significant difference among groups (*P* > 0.05) (Figures 7B,C). In addition, total rearing times and fecal dropping showed no significant difference among groups (*P* > 0.05) (Figures 7E,F).

### Transcranial direct current stimulations slightly improved working memory in T-maze task

**FIGURE 8:**
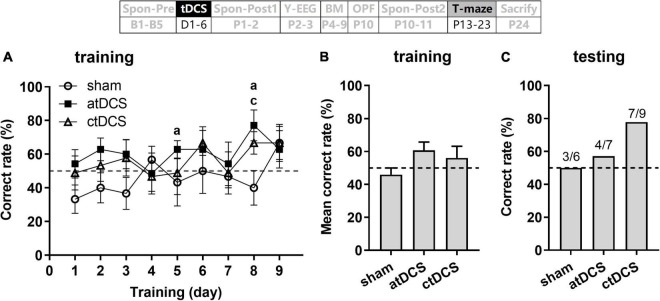
Effects of tDCS on spatial working memory in T-maze task. Correct rate during 9 days training session **(A)**, mean correct rate during this session **(B)**, and correct rate during the testing session **(C)**. Dashed lines indicate chance level (50%). ^a^*P* < 0.05 compared for atDCS and ^c^*P* < 0.05 compared for ctDCS with chance level. The time point of the task relative to tDCS was showed (upper panel).

Combine ANOVA-R revealed no significant change for the correct rate with the training days [*F*(8,152) = 1.56, *P* = 0.14] (Figure 8). However, when compared with the chance level (50%), both atDCS and ctDCS showed significant difference on some days [One-Sample *T*-test; atDCS: *t*(6) = 2.47 and 2.96 for day 5 and 8, respectively, *P* < 0.05 for both; ctDCS: *t*(8) = 2.50, *P* < 0.05 for day 8] (Figure 8A). The difference from the chance level was not significant for any day in sham group (*P* > 0.05 for all days). For atDCS group, the mean correct rate was slightly higher than the chance level [*t*(6) = 2.08, *P* = 0.08] (Figure 8B). During testing day, the ctDCS group (77.8%, 7/9) showed higher correct rate than the sham group (50%, 3/6) (Figure 8C).

### No transcranial direct current stimulations effect on total Aβ concentrations of hippocampus in Alzheimer’s disease mice

**FIGURE 9:**
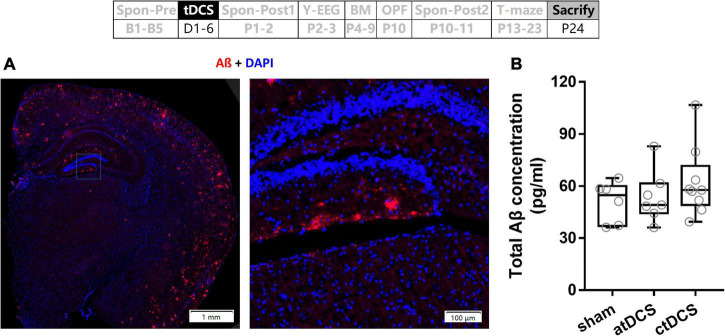
Effects of tDCS on total Aβ concentration. **(A)** An example of Aβ plaques in AD mouse. **(B)** Total Aβ concentrations of hippocampus were compared among tDCS groups. The time point of the measurement relative to tDCS was showed (upper panel).

The AD mice used in the present study were with Aβ plaques, which distributed throughout cortex and hippocampus as well (Figure 9A). However, total Aβ concentrations showed no significant difference among atDCS, ctDCS, and sham groups (Kruskal–Wallis test; χ^2^ = 1.23, *P* = 0.54) (Figure 9B).

## Discussion

In this study, we mainly found that: (1) both atDCS and ctDCS improved spatial learning and/or memory in AD mice without affecting their general locomotion and anxiety-like behaviors, but the improvements depending on the testing paradigms; (2) both atDCS and ctDCS decreased PFC EEG activity in the delta band and increased that in the gamma band when the animals needed memory retrieval during task performance, and the decreases in the delta band were also observed under spontaneous state; (3) specifically, atDCS increased PFC EEG activity in the alpha band for spontaneous state, whereas ctDCS increased that in alpha-beta band for task performance state; and (4) however, tDCS caused no significant changes in Aβ concentrations of hippocampus in the AD mice. PFC EEG alterations in five frequency bands after tDCS were summarized in Table 1. Our results provide the first behavioral and electrophysiological evidence for that both atDCS and ctDCS benefit brain function in the AD animal models.

**TABLE 1:** Summary of PFC EEG activities in AD mice.

PFC EEG frequency (Hz)	Spontaneous EEG (Post vs Pre)			Task-related EEG (Testing vs Training)			Task-related EEG (Novel vs Other)		
	sham	atDCS	ctDCS	sham	atDCS	ctDCS	sham	atDCS	ctDCS
Delta (2–4)		–	–					–	–
Theta (4–8)			+		–				
Alpha (8–12)		+				+			+
Beta (12–20)			–			+			+
Gamma (20–100)						+		+	+

Task-related EEG: EEG activities during Y-maze performance.

It is intriguingly because the atDCS refers to the application of positive current whereas the ctDCS applies negative current to the target, the reversing the polarity of stimulation may cause opposite effects. However, this is not always the case (Das et al., 2016). For example, both atDCS and ctDCS that were applied over the left DLPFC for 10 days could not only improve cognitive function but also reduce P300 latency in AD patients, and the positive effects of the stimulations persisted for 2 months (Khedr et al., 2014). Recently, tDCS, regardless of polarity, was showed efficacious in modulating low-frequency fluctuations of the brain activity in healthy participants, which implies that both atDCS and ctDCS can strengthen global and local brain activity (Ren et al., 2021). In addition, both atDCS and ctDCS were reported to modulate neurogenesis and microglia activation in normal wild-type mouse brain (Pikhovych et al., 2016). The study also suggested multifaceted mechanisms mediating the action of tDCS such as immunomodulation and neurogenesis (Pikhovych et al., 2016). Therefore, future studies are required to confirm if there exists a similar mechanism involved in mediating the actions of atDCS and ctDCS.

In this study, atDCS was found to improve spatial learning and memory and slightly improve spatial working memory in the AD mice, which are in accordance with most clinical studies indicating the beneficial effect of atDCS on physiological and pathological aging-associated cognitive decline (Hsu et al., 2015; Nardone et al., 2015; Prehn and Floel, 2015; Chang et al., 2018). For example, daily atDCS for 6 months improved global cognition that was measured by Mini-Mental State Examination and language function and prevented decreases in executive function in AD patients (Im et al., 2019). Recently, atDCS was reported to enhance spatial working memory in MCI patients (Stonsaovapak et al., 2020). In addition, our data are consistent with some previous studies that atDCS could enhance spatial learning and memory at the early stage of AD mice (Luo et al., 2020) and in a rat model of AD with Aß injected into the bilateral hippocampus (Yu et al., 2015; Yang et al., 2019).

For ctDCS, there are only a few studies performed on physiological and pathological aging processes. The studies showed that ctDCS had little effect on healthy elderly (Harty et al., 2014; Cespon et al., 2017; Adenzato et al., 2019; Ljubisavljevic et al., 2019), but could promote functional neural modulations and improve cognition in AD patients (Khedr et al., 2014; Cespon et al., 2019). In fact, a recent study showed decreased cortical excitability as measured by global mean field power correlated to impaired executive functioning in older adults (Cespon et al., 2022). Considering that ctDCS tends to reduce cortical excitability, it is reasonable that only a few studies were carried out or little effect was observed on cognition for the healthy aging process. However, for the pathological aging process of AD, there are studies indicating neural hyper-excitability in animal models of the disease (Palop et al., 2007; Hall et al., 2015; Scala et al., 2015). Thus, ctDCS may reduce the aberrant cortical excitability and thereby restore cognitive functioning. In accordance with the speculation, our current study provides evidence for the positive effect of ctDCS on cognition in the AD animals.

In addition, we found that tDCS could reverse the situation of slower EEG activity in the AD brain, mainly reflected by decreased delta activity and increased gamma activity in the PFC of animals either under spontaneous or task-related state. Compared with behavioral studies, there are limited EEG studies of tDCS in the AD process. It has been suggested that atDCS can affect the pattern of EEG activity in AD patients, but this requires a more protracted intervention (Marceglia et al., 2016; Gangemi et al., 2020). For slow EEG activity such as the delta, in MCI patients and the patients with temporal lobe dementia or consciousness disorders, atDCS was showed to reduce the delta and/or theta activities (Ferrucci et al., 2018; Mensen et al., 2020; Kim et al., 2021). One of these studies also showed that the cognitive improvements after atDCS significantly correlated with the time course of the slow EEG oscillations (Ferrucci et al., 2018). It was hypothesized that the reduced slow EEG activity might be necessary for the recovery of neural function (Malkani and Zee, 2020; Mensen et al., 2020). We have also reported that the decreased delta activity could explain the improvement in spatial cognition in AD mice (Fu et al., 2019; Zhou et al., 2021). For fast EEG activity such as the gamma, there were reports showing that the task-evoked gamma activity was positively correlated with cognitive abilities in humans (Santarnecchi et al., 2016). Transcranial alternative current stimulation (tACS) at gamma band improved cognitive function in MCI patients (Kim et al., 2021), and visual stimulation at gamma band attenuated Aß plagues in a mouse model of AD (Iaccarino et al., 2016). In addition, atDCS could modulate fronto-parietal high-frequency coherence or temporo-parietal theta-gamma coherence, resulting in improvements on working memory (Marceglia et al., 2016; Jones et al., 2020). Together, these studies suggest that tDCS may modulate both slow and fast EEG activities and then help improve cognition. Our data provide direct evidence for that tDCS including both anode and cathode can reduce EEG slowing in the AD brain, which may further lead to the improvement of spatial cognition.

Specifically, atDCS increased PFC EEG activity in the alpha band for spontaneous state. Resting alpha activity plays an important role in the coordination of brain networks. The lower alpha seems to be related to the globally long-range connectivity, whereas upper alpha may be related to the transmission of sensorimotor information as well as to semantic memory retrieval (Tatti et al., 2016). The continuous decreases in alpha activity and coherence were suggested to be biomarker for progressing from healthy aging, to MCI, and to AD (Tatti et al., 2016). It was reported that the increases in alpha-beta activities after atDCS were correlated with the improvement of working memory in AD patients (Marceglia et al., 2016). Thus, the effect of atDCS on alpha band in this study was in accordance with the previous reports.

On the other side, ctDCS specifically increased PFC EEG activities in alpha-beta band for task performance state. Notably, compared with atDCS, ctDCS induced more EEG changes in the AD mice, reflected by changes in more frequency bands during task performance and by changes in other cortical regions besides PFC. tDCS can modulate cortical excitability during and after stimulation, with atDCS increasing and ctDCS decreasing excitability (Nitsche et al., 2003). In AD patients, slight tendencies between enhanced working memory and increased P200 after ctDCS were observed, but no significance was found after atDCS (Cespon et al., 2019). In addition, ctDCS induced more neurogenesis from the subventricular zone than atDCS in normal mouse brain (Pikhovych et al., 2016). However, further studies are needed to confirm if ctDCS has a more remarkable effect than atDCS on the AD brain.

In the present study, we did not observe significant changes of Aβ content after tDCS. The finding is consistent with a previous study showing that atDCS (50 μA, 0.0325 cm^2^, 20 min/day) for 15 days led no significant changes on APP and tau levels in AD mice (3xTg; 6–7 months) (Gondard et al., 2019). However, in another study, atDCS (150 μA, 0.0314 cm^2^, 30 min/day) for 5 days significantly decreased Aβ levels in AD mice (APP/PS1; 6 months) (Luo et al., 2020). In our study, both atDCS and ctDCS were given at 300 μA (0.096 cm^2^) for 20 min/day for 5 days. We calculated the stimulation current densities for these studies. The value was higher in the study showing significant decreases of Aβ levels after tDCS (4.78 mA/cm^2^) than those showing no obvious changes (1.54 and 3.12 mA/cm^2^). Thus, further evidence is required to confirm if the pathological hallmarks of the AD can be attenuated by increasing the current density of tDCS. It is also possible that the effect of tDCS on AD brain does not involve with a clearance of Aβ, since the clearance of Aβ did not prevent progressive neurodegeneration in AD patients (Holmes et al., 2008).

The paradigms used in this study included Y-maze, Barnes maze and T-maze tasks, separately for testing spatial memory (short-term), learning and memory (long-term) and working memory. We found that the cognitive improvements of tDCS depended on the testing paradigms. For example, the improvement of atDCS was found by Barnes maze task, and that of ctDCS was observed by Y-maze task. It was noted that the effect of ctDCS, which has been rarely reported previously in AD animals, was found by the Y-maze task. The task does not require the animal to learn rules and is based on the innate tendency of rodents to explore novel environments (Dellu et al., 2000). Thus, considering the inconsistent results previously reported, it would be necessary, in the future, to combine two or more paradigms to test the effect of tDCS, especially for ctDCS.

In summary, we found that tDCS including anode and cathode fasted EEG activity and improved spatial cognition in APP/PS1 double transgenic mice. Our study highlights the potential clinical use of tDCS to improve cognitive disorders and restore neural network activity in AD. Importantly, more attention should be paid to the research of improving EEG activity through ctDCS to improve cognition.

## Data availability statement

The raw data supporting the conclusions of this article are available on request to the corresponding author.

## Ethics statement

The animal study was reviewed and approved by Animal Care and Ethics Committee of Kunming University of Science and Technology (approval no. 20190001).

## Author contributions

YF, ZM, and TT contributed to conception and design of the study. MD, DY, and YZ performed the experiments. MD, ZM, ZC, and YF performed the statistical analysis. MD and YF wrote the draft of the manuscript. All authors contributed to manuscript revision, read, and approved the submitted version.
